# Emerging Developments in Human Induced Pluripotent Stem Cell-Derived Microglia: Implications for Modelling Psychiatric Disorders With a Neurodevelopmental Origin

**DOI:** 10.3389/fpsyt.2020.00789

**Published:** 2020-08-11

**Authors:** Bjørn Hanger, Amalie Couch, Lawrence Rajendran, Deepak P. Srivastava, Anthony C. Vernon

**Affiliations:** ^1^Department of Basic and Clinical Neuroscience, Institute of Psychiatry, Psychology and Neuroscience, Maurice Wohl Clinical Neuroscience Institute, King’s College London, London, United Kingdom; ^2^UK Dementia Research Institute, Institute of Psychiatry, Psychology & Neuroscience, King’s College London, London, United Kingdom; ^3^MRC Centre for Neurodevelopmental Disorders, King’s College London, London, United Kingdom

**Keywords:** microglia, neuroinflammation, human induced pluripotent stem cells, neurodevelopmental disorders, schizophrenia, autism

## Abstract

Microglia, the resident tissue macrophages of the brain, are increasingly implicated in the pathophysiology of psychiatric disorders with a neurodevelopmental origin, including schizophrenia. To date, however, our understanding of the potential role for these cells in schizophrenia has been informed by studies of aged *post-mortem* samples, low resolution *in vivo* neuroimaging and rodent models. Whilst these have provided important insights, including signs of the heterogeneous nature of microglia, we currently lack a validated human *in vitro* system to characterize microglia in the context of brain health and disease during neurodevelopment. Primarily, this reflects a lack of access to human primary tissue during developmental stages. In this review, we first describe microglia, including their ontogeny and heterogeneity and consider their role in brain development. We then provide an evaluation of the potential for differentiating microglia from human induced pluripotent stem cells (hiPSCs) as a robust *in vitro* human model system to study these cells. We find the majority of protocols for hiPSC-derived microglia generate cells characteristically similar to foetal stage microglia when exposed to neuronal environment-like cues. This may represent a robust and relevant model for the study of cellular and molecular mechanisms in schizophrenia. Each protocol however, provides unique benefits as well as shortcomings, highlighting the need for context-dependent protocol choice and cross-lab collaboration and communication to identify the most robust and translatable microglia model.

## Microglia—A Short Introduction

Microglia are the primary immunocompetent cells of the central nervous system (CNS). In the adult brain they are thought to play key roles in shaping the local tissue response to injury, infection, damage, and in maintaining CNS homeostasis (1). Microglia also are also increasingly appreciated to play a key role in brain development (2). As a result, functional disruption of these cells has been linked to the pathogenesis of a variety of brain disorders (3). Following their description in the early 20^th^ century by Del Río Hortega (4), microglia have conventionally been studied *en bloc* using low-resolution *in vivo* positron emission tomography (PET) and *post-mortem* tissue from human and rodent brains, but evidence of the heterogeneous nature of these cells is accumulating (5). Notably, until recently, *in vitro* studies of microglia have relied on immortalized cell lines derived from either mouse or human sources. These immortalized microglia cell lines such as, mouse BV2 or human SV40 cell lines, whilst useful for generating hypotheses for further study are no longer considered representative of primary microglia, since they do not express core microglial signature genes (6–8). The great majority of data and hence our understanding of microglia biology also comes from rodents and it is unclear how these data generalizes to humans (9). Collectively, these points suggest a need for a flexible and reliable human *in vitro* model system, with which to study microglia biology in the context of health and disease including neurodevelopmental stages. Access to human primary tissue, particularly foetal tissue, is however very limited. Furthermore, it is unclear to what extent microglia harvested from peri-lesional areas during surgical resections in the adult brain may reflect “normal” microglia. In this review, we address the potential of microglia derived from human induced pluripotent stem cells (hiPSCs) as a potential candidate model system to address this gap. In doing so we first describe microglia, including their ontogeny and heterogeneity and consider their role in brain development. We then provide an evaluation of published protocols for differentiating microglia from hiPSCs and their potential use as a robust *in vitro* human model system to study these cells and characterize them in the context of health and psychiatric disorders with a putative neurodevelopmental origin, including schizophrenia (SZ). The potential for hiPSC-derived microglia in modelling age-related neurodegeneration has been recently reviewed elsewhere (10).

## Microglia Ontogeny

**Figure 1:**
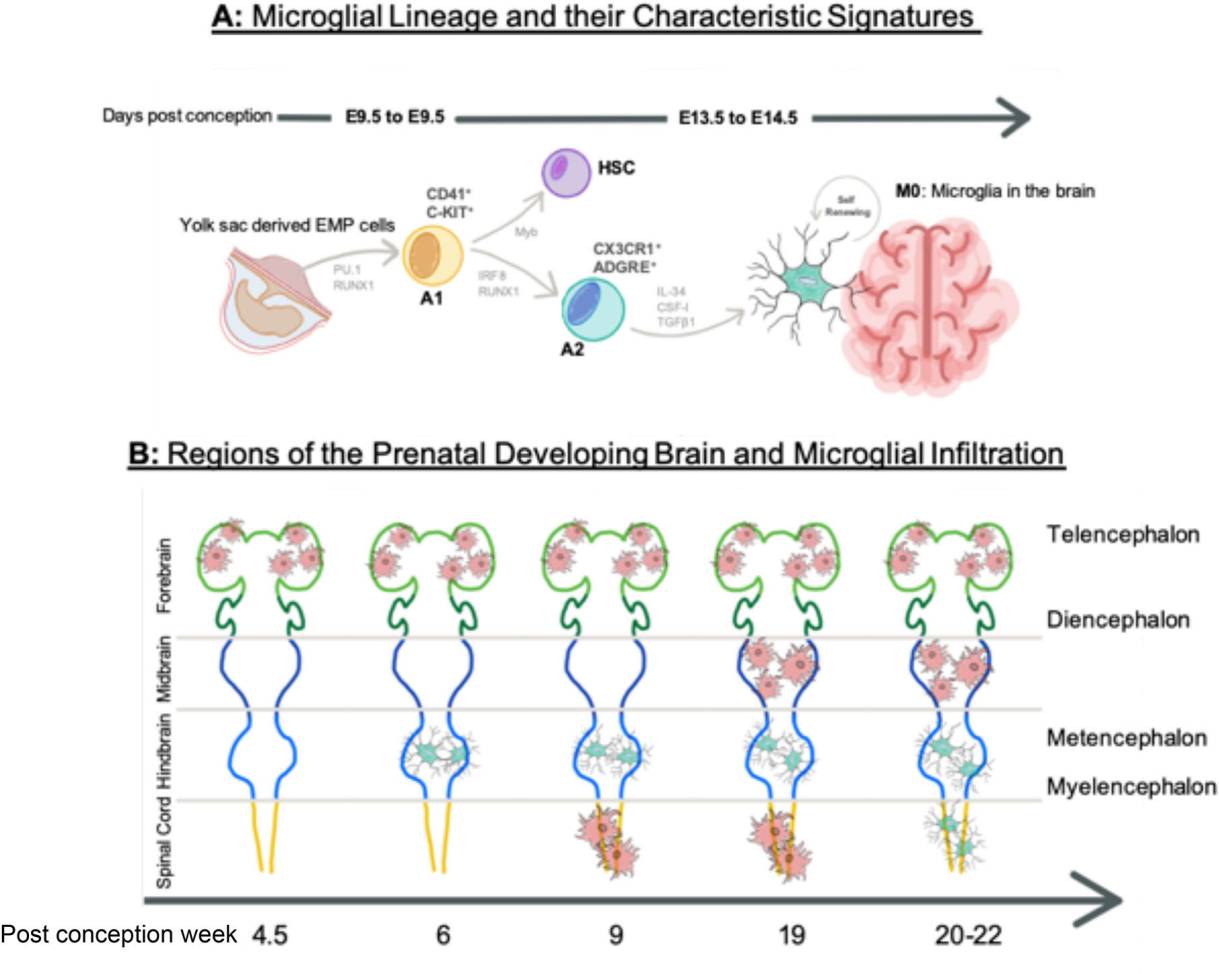
Microglial Ontogeny. **(A)** The mesodermal theory of microglial origin, where erythroid progenitor cells (EMP) are directed through progenitor stages A1 and A2 by transcription factors PU.1, RUNX1, and IRF8 during E8.5–9.5. They are independent of Myb influence, which direct hematopoietic stem cell (HSC) lineage. Progenitor microglia assemble at M0 stage in the brain until E14.5, where they self-renew. **(B)** Microglia infiltration into the developing brain at different regions over the course of gestation. Amoeboid state microglia are shown in red and ramified microglia are show in blue. Time is shown in post-conception weeks. Image in **Figure 1** **(A)** contains a modified form of this image: https://molecularbrain.biomedcentral.com/articles/10.1186/s13041-017-0307-x/figures/1 by (21), used under CC BY 4.0: (http://creativecommons.org/licenses/by/4.0/), colours changed from original and new elements added. Image in **Figure 1** **(B)** contains a modified form of this image http://commons.wikimedia.org/wiki/File:EmbryonicBrain.svg by Nrets, used under CC-BY-SA: http://creativecommons.org/licenses/by-sa/2.5/ colours changed from original.

The origin of microglia spans two major theories, arguing whether the microglia precursors originate in the mesoderm or the neuroectoderm. The neuroectoderm theory places microglia in the same lineage as astrocytes and oligodendrocytes (11–14), while the mesoderm theory suggests a hematopoietic yolk sac (YS) origin (15–17). Critically, following lineage tracing studies it is becoming increasingly evident that under normal conditions the latter YS origin is the sole source of microglia during development (18). This also suggests that when compared to non-CNS macrophages, microglia uniquely derive from tissue-resident erythromyeloid-derived macrophage precursors, which infiltrate into the developing brain parenchyma through blood vessels between rodent embryonic day (E) 8.5–9.5 (18, 19). A key characteristic of this lineage is Myb (MYB Proto-Oncogene, Transcription Factor)-independence, a transcription factor which is required for non-YS macrophage and monocyte development, since expression of other key transcription factors, such as PU.1 and Irf8 respectively regulates microglial fate determination and influences microglial progenitor survival (15, 20). A schematic diagram of microglia maturation from the YS is shown in **Figure 1A**.

The appearance of macrophage precursors predates neurogenesis, making microglia one of the first residents of the brain (16). Ramified microglia-like cells are observed at post-conception (pc) week 6 in the hindbrain, following the peak of YS haematopoiesis (22). Microglia in an amoeboid-like state are however, observed in the intermediate, telencephalic ventricular, and marginal zones at PC week 4.5. Functionally, these foetal stage microglia gain the ability to detect and react to local environmental changes in the mouse at E16.5 (approx. PC week 8 in humans) and are characterized by high expression of several genes termed the microglial “sensome” (23, 24). In humans, at PC week 9, amoeboid–like microglia are also observed in the spinal cord before their appearance with similar morphology in the mesencephalon around PC week 19, reaching peak density and displaying a ramified morphology, around the time oligodendrocyte and astrocyte precursors appear around PC week 20–22 (25–27). To summarize these data, **Figure 1B** highlights the appearance microglia in different CNS regions by PC week. Whilst data from human brain material exists, most accumulated data on microglial infiltration into the brain is predominantly inferred from rodent models. Species differences in microglia ontogeny are however reported to exist and further studies are required to substantiate if these exist and how important they may be (9).

## Heterogeneity of Microglia

Following the degeneration of the yolk sac (PC week 9), microglia exploit their capacity for clonal expansion to increase and then maintain the brain population at a steady state during development. In this period, neurogenesis and neural migration also occurs, creating local cues that influence the form and function of microglia. Data from rodents suggest that in turn the microglia may well then actively play a role in shaping brain connectivity by several means, including modulation of axon growth cone guidance and synaptogenesis (28–30). This initial neuron-microglia contact may represent the beginning of a long-lasting diversification of microglia into brain region-specific phenotypes. Consistent with this view, foetal murine microglia are highly heterogeneous as indexed by single cell RNA sequencing (31). Such diversity of microglia has been observed as early as mouse E18.5. This also corresponds to a period of male/female differentiation, with the acquisition of sexually dimorphic transcriptomic signatures characterized by an increase of differentially expressed genes (DEGs) primarily on the X and Y chromosomes, which only becomes more different with age and could underpin reported sex differences in microglia form and function (24, 32). Although there is considerable similarity between human and murine microglia, it is notable that several genes that are not part of the mouse microglia signature are highly expressed in human microglia. These include, for example, genes associated with the cell cycle (*TAL1*), and proliferation (*IFI16*) (31, 33, 34). To further emphasise the potential relevance of species differences, in **Table 1** we highlight a number of species differences in microglia that may be of relevance to psychiatric disorders. It is unknown however, to what extent these apparent species differences influence the cellular functions of human microglia cells during development, reinforcing the need for further studies in human model systems.

**Table 1:** Overview of species differences between human and mouse microglia of potential relevance to using human iPSC-derived microglia for studying neuropsychiatric disorders.

Phenotype	Species differences of potential relevance to studying microglia involvement in human psychiatric disorders with a neurodevelopmental onset	References
**Microglia turnover and maintenance**	Rates of turnover for rodent microglia vary from 0.05 to 0.7% depending on the method used Human microglia may be longer-lived with slower turnover relative to the lifespan of the host species although chimeric model data suggest fast turnover and proliferation of human microglia in the neonatal rodent brain	Lawson et al. (35); Askew et al. (36) Reu et al. (37); Xu et al. (38)
**Microglia gene expression signature (homeostatic state)**	High correlation in gene expression signature between microglia isolated from human *post-mortem* and surgically resected brain tissue (*r* = 0.94) Only >50% overlap to rodent microglia with species specific differences in gene expression (either unique in mouse or human)	Galatro et al. (33); Gosselin et al. (39) Dubbelaar et al. (40)
**Microglia diversity along spatial and developmental dimensions**	Single cell RNA sequencing confirm that rodent microglia show regional and time dependent heterogeneity, which is maximal during development Human microglia show similar heterogeneity but formal comparisons to mouse datasets are lacking, qualitatively a partial overlap is reported	Grabert et al. (41); Hammond et al. (42) Masuda et al. (43)
**Response to interferon-γ / LPS stimulation *in vitro***	Rodent microglia become rounded/amoeboid, retract processes, increasing TSPO and iNOS/Arginase1 expression Human microglia in contrast extend processes, becoming bipolar, decrease TSPO expression and iNOS/Arginase1 is not induced	Healy et al. (9); Nakamura et al. (44); Owen et al. (45)

TSPO, translocator protein; iNOS, induced nitric oxide synthase. Additional references not in main text: (33–35, 38, 40, 42, 43).

While microglia in the adult rodent brain are potentially much less diverse as compared to the developing brain, there is some evidence that apparent regional transcriptional heterogeneity is maintained, which may be further enhanced during ageing (41). For example, Grabert and colleagues (40) provide evidence for such heterogeneity in the adult mouse brain based on microarray gene expression mapping of microglia isolated from fore-, mid-, and hindbrain regions (41). Basal ganglia specific signatures from anatomical features to transcriptome differences dependent on local cues have also been shown in the mouse brain (28). These molecular observations are however consistent with earlier *post-mortem* work detailing regional diversity in microglia density and morphology in the rodent brain (46, 47). Translating these findings to humans, mass cytometry analysis of surface protein markers expressed on microglia suggests that subventricular zone microglia in the human brain represent a distinct population as compared to other brain regions, although phenotypic variability between donors was also reported (48). A deeper analysis of this dataset suggested additionally that the sub-ventricular zone (SVZ) and thalamus (THA) contain similar microglial phenotypes, not observed in the other brain regions examined (48). Moreover, the temporal and frontal lobes are enriched in yet other distinct microglia phenotypes (48). Finally, the microglia profile in the cerebellum (CER) also appeared to be quite distinct from the other brain regions examined (48). These data, particularly for the cerebellum are in good agreement with extant mouse transcriptional data, which also suggests a marked difference between cerebellar and forebrain microglia phenotypes (41). Building on these data, single-cell RNA sequencing work, combined with mass cytometry confirms distinct transcriptional profiles of microglia within human temporal lobe tissue biopsies from epilepsy and glioma patients (49). At the cellular level, hippocampal microglia density differences are reported in neurodegenerative disease samples (50). Transcriptional profiling studies have also further distinguished this heterogeneity between grey-white matter specific profiles (49, 51) and specific time-and region dependent subtypes (43). The generation of such regional microglia subtypes may be reliant on unique molecular programs induced by exposure to specific regional cues. For example, Kana et al. (52) demonstrate that cerebellar microglia identity appears to be driven by high local levels of Colony Stimulating Factor-1 protein (CSF-1), with only forebrain microglia remaining intact when CSF-1 was depleted genetically from nestin-positive cells (52). Consistent with the idea of diverse molecular machinery, homeostatic microglia density is retained in a space- and time-dependent manner even after acute chemical ablation (53). Hence both intrinsic and local signals must contain information to repopulate to region-specific densities of microglia. The functional significance of this heterogeneity is currently unclear. On the other hand, such heterogeneity would be consistent with the evolutionary pressure for innate immune cells to be able to respond flexibly to different pathogens or tissue injuries. Regardless, it is evident that local cues play important roles in defining microglia phenotype at both the transcriptional and protein expression levels. At present, the extant human data on microglia heterogeneity is based on *post-mortem* samples collected from adult individuals, with little or no information available from foetal or embryonic brain tissue. Whilst mouse models attempting to characterize these early periods provide extremely useful data, the potential for species-related heterogeneity is still a challenge for the field. Accordingly, a human-relevant model system, where microglia subtypes in the context of the foetal, developing brain can robustly be characterized under basal and disease conditions remain a pressing need.

## Microglia “Activation” and Neuroinflammation

Brain-resident microglia are part of the innate immune system, which provides the brain with a rapid, non-specific first line of defense against pathogens. This should be distinguished from the adaptive immune system, which primarily involves T-lymphocytes and is slower and antigen specific (54, 55). Microglia activation is thus classified as a shift away from homeostasis and activation of a defense response, which occurs downstream of different stress signals, collectively know as “pathogen-associated damage patterns” (PAMPs) and “damage associated patterns” (DAMPs) that are recognized *via* pattern recognition receptors (PRRs) (54, 56). These PRRs expressed in microglia include Toll-like receptors (TLR), which sense components of bacterial (TLR4) or viral (TLR3, 7, 8) pathogens. Hence LPS (a TLR4 agonist) is commonly used as a tool to induce microglia activation *in vitro* (57), *in vivo* in experimental animals (58) and healthy humans (59). Such microglial activation is characterized by a shift in morphology, a shift to anaerobic metabolism, increased reactive oxygen species (ROS) production, increased synthesis and release of cytokines and chemokines, microglial clustering, and/or migration and phagocytosis. Based largely on *in vitro* studies, following activation, for example, by LPS, microglia have been categorized into either “protective” (termed M2) or “toxic” (termed M1) states (60). This classification however has recently been challenged, since *in vivo* studies clearly show that microglia in the rodent brain can express genes associated with both M1 and M2 states simultaneously (3). Furthermore, it is unlikely that similar transcriptional profiles will be adopted in heterogeneous microglia across different brain regions (49), or where the microglia may be hypersensitive to stimulation, described as “primed” microglia, for example, following early developmental insults such as childhood CNS or severe systemic infection, or as a function of ageing and age-related disorders (55, 61, 62). Nonetheless, pro-inflammatory stimulation may be used to assess several aspects of microglia activation in the context of health and disease, including phagocytosis, cytokine expression, reactive oxygen species production, synaptic pruning, and neuronal survival, which may all be viably assayed *in vitro*, which can form the basis for *in vivo* validation (57, 63). Different pro-inflammatory stimulants may however yield both common and distinct phenotypes *in vitro* in such assays and thus choice of stimulation paradigm or testing of multiple stimulation contexts is essential to generate meaningful results (64). Recent studies using human microglia-like cells (MGLs) differentiated from hiPSCs demonstrate that these cells have a clear activation response following stimulation with lipopolysaccharide (LPS) and interferon-γ, but also that this may be influenced by disease-relevant mutations, for example in triggering receptor expressed on myeloid cells-2 (*TREM2*) (57, 63). Notably however, this activation response differs across species (**Table 1**). These data suggest a view that studying the response of hiPSC-microglia derived from individuals with schizophrenia following stimulation with different PAMPs/DAMPs may be useful. Specifically, this could help to assess if there is a general difference in microglial activation as a function of SZ diagnosis or whether this is only relevant for particular individuals, depending on their genetic and/or environmental risk exposures. Studies on the role of the inflammasome may be particularly relevant in this context. Inflammasomes are multi-protein complexes that form following stimulation of PRRs by PAMPs/DAMPs in microglia of which, the NLRP3 inflammasome is the best-described (65). Upon stimulation, the sensor molecule NLPR3 recruits pro-caspase-1 *via* the adaptor molecule apoptosis-associated speck-like protein (ACS), resulting in cleavage of the cytokine precursors pro-IL-1β and pro-IL-18 into mature IL-1β and IL-18 and their subsequent release from microglia (65). This is relevant in the context of this review, since there is evidence for the involvement of both IL-1β and IL-18 in schizophrenia pathophysiology. For example, elevated serum and *post-mortem* brain IL-1β levels have been reported in SZ and are associated with both symptom severity and disease progression (66, 67). Hypothetically at least, such phenomena could occur downstream of NLPR3 inflammasome activity in activated microglia (55). To date, we are not aware of any published studies on inflammasomes that have been carried out in MGLs derived from patients with SZ or other psychiatric disorders, using a protocol that recapitulates appropriate human microglia ontogeny. Given the predicted key roles of microglia in brain development (as described in the next section) and the links between SZ risk and early developmental insults such as maternal, childhood CNS, or severe systemic infection (55), this is a key gap in our knowledge that remains to be addressed.

## Roles of Microglia in Brain Development

In addition to carrying out fundamental immune and homeostatic responses, microglia play two major roles in brain development; the phagocytosis of unwanted neurons and modulating synaptic connections. The latter occurs in the dual context of not only promoting synapse formation but also in synapse elimination, which may occur in a time and region-specific manner (68). For example, through the production of reactive oxygen species (ROS), which is linked to their expression of DNAX-activation protein 12 (DAP12) and CD11b, microglia promote the engulfment of cerebellar Purkinje neurons and hippocampal neurons during development (69, 70). Moreover, CSF-1 deficiency and the subsequent alteration of cerebellar microglia are reported to be associated with reduced numbers of Purkinje cells, altered neuronal function, and defects in motor learning and social novelty interactions (52). Microglia regulation of neuronal progenitor pools is also retained from development to adulthood, with there being evidence of homeostatic phagocytosis in the subgranular zone neurogenic niche (71).

The functional role of microglia in regulating synaptic connections was first suggested by Blinzinger and Kreutzberg following *in vitro* experiments (72). Recent studies have now provided *in vivo* evidence of the contact between microglia and synaptic structures, describing both filopodia formation and elimination in an activity and complement dependent manner in the developing mouse cortex (73–75). The nature of these interactions appears to be both region- and time-dependent. For example, filopodia formation following microglia contact appears to occur at early periods of synaptogenesis in the developing somatosensory cortex at postnatal day 8–10, possibly driven by neuronal activity in this period (76). Microglia have also been posited to remodel or refine mature existing synapses through their elimination. Studies in the mouse brain provide evidence for the engulfment of synaptic material in an activity and complement dependent manner, which is exacerbated in mice with pathology associated with neurodegeneration, such as amyloid-β plaque formation (74, 77). Moreover, loss of microglia-neuron cross talk *via* genetic deletion of the fractalkine receptor (CX3CR1) also negatively impacts on putative synaptic pruning by microglia leading to abnormal brain development and the emergence of impairments in brain connectivity and social behavior in the adult animal (73, 78). In contrast, synaptic plasticity in the visual cortex does not appear to be affected by CX3CR1 deletion (79, 80). Hence the regional specificity of putative microglia-mediated synapse elimination remains to be established. The precise nature of the interaction between microglia and neurons leading to remodelling of synapses is also suggested not to represent engulfment *per se*, but may be best described as “trogocytosis” (81, 82).

Translating these data to humans, induced microglia (iMG) generated from peripheral blood mononuclear cells (PMBCs) engulf synaptic material *in vitro*, which is enhanced in iMG from individuals with a diagnosis of SZ (7, 83). There are however no studies modelling human microglia-synapse interactions *in vitro* that incorporate microglia with the correct YS ontogeny, which will be helpful to confirm the aforementioned exciting findings from iMG. Moreover, the evidence for engulfment of synaptic material by microglia in both rodent and human models is principally based on imaging of fixed tissues, whereas imaging dynamic microglia-synapse interactions would be desirable. Finally, the precise molecular mechanisms driving these microglia-neuron interactions remain to be characterized in detail. For example, if microglia do “prune” synaptic connections during development, what are the molecular signals that regulate this process? Whilst CX3CR1 and the complement system are clearly leading candidates based on schizophrenia genetics, much work remains to be done in this area. In particular, little has been done to assess the impact of environmental risk factors, linked to the innate immune system that are associated with increased risk for SZ and other psychiatric disorders. For instance, as alluded to in the previous section, early developmental insults following maternal or childhood brain and/or severe systemic infection are associated with increased risk for SZ in the affected offspring/children (54, 55). In this context, data from a mouse model of maternal immune activation (MIA) provides evidence for increased spine density in the hippocampus of MIA-exposed male offspring early in development (post-natal day 15) and decreased expression of CX3CR1 (84). In contrast, a loss of post-synaptic proteins has been reported in the hippocampus of male MIA-exposed offspring in the pubescent period (post-natal day 35), which was maintained into adulthood (post-natal day 90) (85). Other groups have also reported elevated levels of complement factors involved in synaptic pruning, namely, C1q and C4 in rodent offspring exposed to MIA *in utero* (86, 87). The data on microglial activation in rodent models of MIA is however, by no means unequivocal, with evidence for both persistent microglial activation, or no overt microglial activation, as reviewed elsewhere (88). Collectively, these data suggest that part of the risk mechanism that links MIA to psychiatric disorders, including SZ, may involve abnormal neuron-microglia interactions and synaptic pruning, which may differ depending on the neurodevelopmental stage examined. A flexible, robust, *in vitro* model of human microglia-synapse interactions, particularly one amenable to high speed, multi-photon live imaging would be extremely useful to investigate this further alongside the effects of genetic risk factors for SZ on microglia-synapse interactions. Ideally, as already stated, such a model would benefit from microglia that show correct human ontogeny, as evidence for microglia generated from hiPSCs (57). Before considering this question in more detail however, it is important to first briefly reflect on the evidence base for microglial activation in SZ.

## Evidence for Microglial Activation in Psychiatric Disorders With a Neurodevelopmental Origin

SZ is a complex debilitating neurological disorder affecting approximately 1% of the population, presenting with positive and negative symptoms, cognitive dysfunctions, and reduced psychosocial function. The exact causes of SZ remain elusive, but it is highly heritable, albeit with a complex, polygenic architecture. Highly penetrant rare variants, particularly copy number variants (CNVs) do however exist that are associated with a significantly increased risk for SZ. For example, 22q11.2 deletion syndrome (DiGeorge Syndrome) is associated with a 20-fold increased risk for SZ in carriers (89). Peripheral neuroinflammation, characterized by raised circulating pro-inflammatory cytokines is a hallmark of several psychiatric disorders, including SZ, (90). In the CNS, there are also converging lines of evidence from genetics, neuroimaging, and *post-mortem* studies for microglial activation in these disorders, although this evidence is by no means unequivocal (91). It remains unclear however, to what extent microglial activation is causative for psychiatric symptoms, or simply a homeostatic defence response to the diseased brain state. In support of the latter view, isolation of microglia from *post-mortem* brain tissue of individuals with bipolar disorder suggests these cells are not activated (92). Notably, such data for SZ brain tissue is currently lacking in the literature. On the other hand, consistent with the key roles that microglia are thought to play in shaping brain development, gain or loss of microglia function during critical periods of brain development could plausibly lead to abnormal neural circuit formation and the later emergence of psychopathology. In support of this view, there is strong genetic evidence for a link between increased numbers of complement C4A alleles and higher risk for SZ (93). Notably, C4A knockout leads to abnormal synaptic pruning in mice (93). Furthermore, while not establishing a causal relationship between SZ risk variants and synaptic pruning, Sellgren and colleagues reported that the C4 risk variant of SZ is associated with an increased capacity of blood-derived iMG to phagocytose synaptic material *in vitro* (83). It would however be desirable to confirm these exciting findings using human microglia that are generated with an authentic, yolk-sac ontogeny, as already alluded to in the preceding section. Such a model would also be useful to address a number of other gaps in our knowledge mentioned throughout this review. It is now possible to generate hiPSC-derived microglia and cortical neurons in a functional co-culture system, within which the microglia transcriptionally show resemblance to foetal microglia (57). Yet to date, there are no published reports describing a phenotype in hiPSC-derived microglia from individuals with psychiatric disorders, including SZ, with the primary focus having been to date on neurodegeneration (10). Importantly however, the work of Sellgren and colleagues clearly underscores the potential of novel human *in vitro* microglia model system in determining the effect of genetic risk factors for psychiatric disorders on microglia phenotypes, which may also be extended to environmental risk factors such as MIA including how this interacts with genetic risk. We therefore consider this potential in the next sections in more detail.

## The Potential and Limitations of hiPSC-Derived Microglia Models for Modelling Psychiatric Disorders

As already stated, most research on microglia in the context of psychiatric disorders, such as SZ utilizes human *in vivo* neuroimaging methods [e.g. radioligand targeting of Translocator Protein (TSPO) expressed by microglia detected by PET] or is heavily reliant on analysis of *post-mortem* tissue from human brain banks and rodent *in vivo* and *in vitro* models. Concerns have arisen however over the specificity of TSPO for imaging putative microgliosis *in vivo*, since this protein is also expressed in astrocytes and endothelial cells (94). Furthermore, the results of TSPO PET studies in SZ are by no means unequivocal. Moreover, it is impossible to link changes in TSPO radio-ligand binding in humans to microglial phenotypes *in vivo*, hence we lack a detailed understanding of how a change in TSPO binding as measured by PET relates to microglia functional state and whether this is beneficial or detrimental in SZ compared to healthy controls. *Post-mortem* data, whilst informative, is subject to numerous confounds including age-related changes in controls and prolonged exposure to psychotropic medications. For example, chronic antipsychotic drug exposure is reported to directly affect microglia morphology and density in the rat brain in a time and dose-dependent manner (95, 96). As yet however, we do not understand if this reflects beneficial or detrimental changes, or whether these findings translate to humans (97). Whilst rodent models offer much more experimental scope and flexibility, potential species-specific differences in microglia remains an important, yet weakly addressed issue (9) (see also **Table 1**). Hence, again, the case for a relevant human *in vitro* model to fill this gap is reinforced.

A clear candidate to fill this gap are hiPSC-derived microglia, which offer the potential for a patient-specific model system, with the capacity to study the effects of genetic mutations associated with SZ (and other psychiatric disorders). These cells also have clinical applications including gene therapy, drug testing, and autologous cell replacement therapy. Derivation of neuronal cells from hiPSC has already been demonstrated by many laboratories to successfully capture differences in genotype and phenotype in cells derived from individuals with psychiatric disorders, including SZ, that originate during neurodevelopment (98–102). Yet non-neuronal cells, including microglia remain understudied, despite the aforementioned evidence for their potential involvement in SZ (103).

To date, several protocols have been published in the literature describing the generation of hiPSC-derived microglia-like or macrophage-like cells from human tissues within the last 5 years (see **Table 2**). These protocols all share common advantages in providing high yields of cells and overall, the phenotype of the cells produced appears to be aligned with tissue resident macrophages and brain-localized microglia, albeit perhaps more closely aligned to foetal microglia, as evidenced by transcriptional profiling (57, 63, 106). The majority of published protocols supplement hiPSC with growth factors to specify mesodermal fate, leading to development of primitive haematopoietic progenitors, followed by maturation along the myeloid lineage using specific growth factor cocktails (see **Table 2**) (113). This has led to some contention in the field however, based on debate regarding what constitutes “authentic” microglial ontogeny, as compared to that of peripheral macrophages. As already mentioned in this review, microglia ontogeny is thought to be *via* EMP that arise from the YS in a Myb-independent, but PU.1 and RUNX.1 dependent manner (see **Figure 1A**) (15, 18, 112, 114). Based on these data, recent studies have presented refined protocols that recapitulate a YS-microglia ontogeny, which may be suggested to reflect true microglia-like cells (10, 57, 63). Nonetheless, the debate continues as to which protocol may offer the most “optimal” solution as well as how we should accurately define what is a “true” microglial cell derived from hiPSC.

**Table 2:** Overview of published hiPSC-derived microglia models. While all these protocols can be concluded to produce microglia-like phenotypes, co-culture models that provide cues associated with a CNS environment are the most promising.

Article	Overview of protocol	Notable findings	Notable disadvantages
Almeida et al. (104)	Not described in publication	First to produce hiPSC-microglia	Transcriptomic profile not unlike immortalized microglia cell lines (BV-2) Generated through neuronal rather than myeloid pathway.
Muffat et al. (105)	Embryoid bodies were generated and resuspended in neuroglial differentiation media containing (supplement) with the addition of CSF-1/M-CSF and IL-34	First published study with similar characteristics of fetal primary human and mouse microglia.	Appears to generate a mixed population of cells and is limited to monoculture experiments.
Abud et al. (106)	Microglia differentiation media utilizes neuronal base media DMEM/F-12 + +N2+B27 with small molecules M-CSF, IL-34, and TGFβ-1. An additional maturation media is utilized consisting of CD200 and CX3CL1, which is notably secreted by neurons for the final three days.	Successful transplantation of already ramified microglia within Alzheimer’s disease model mice. Subsequent in vivo evidence shows ability to interact with neurotoxic amyloid β	Requires an isolation step to begin differentiation part of haematopoiesis step, making it highly complex compared to pure single molecule methods. Not authentic YS ontogeny.
McQuade et al. (107)	Proprietary composition of initial hematopoietic differentiation media (STEMdiff hematopoietic kit) for an 11-day period followed by differentiation with IL-34, TGF-β1, and M-CSF/CSF-1. Includes the additional maturation step with CX3CL1 (fractalkine) and CD200 to induce ramification.	Successfully ramify following transplantation in mouse brain. Suggests IDE1 as a small molecule able to replace TGF-β in protocols utilizing this for differentiation.	Describes itself as resembling developmental microglia but does not separate cited fetal vs adult datasets. Not authentic YS ontogeny
Takata et al. (108)	Generation of hematopoietic lineage macrophages terminally differentiated with SCF, IL-3 and CSF-1/M-CSF. Cells then co-cultured with mouse iPSC-derived neurons to further drive towards microglia phenotype	Described the requirement for tissue-dependent cues in order to make cells more microglia-like. Demonstrated potential of modelling infiltrating macrophages during adulthood.	Primary characterization with mouse iPSCs. Not authentic YS ontogeny
Pandya et al. (109)	iPSCs were differentiated on OP9 feeder layers with OP9 differentiation medium (ODM) to myeloid progenitors. CD34+/CD43+ cells were sorted with MACS into myeloid progenitor media with GM-CSF and subsequently passaged and plated in astrocyte differentiation medium (ADM-IMDM base medium + GM-CSF, M-CSF and IL-3) then CD11+ cells were further isolated. Additionally, some experiments used CD39+ microglia sorted from a specific co-culture system with astrocytes.	Utilizes hematopoietic stem cells paired with astrocytes to obtain iPSC-derived microglia. Mouse iPSC-derived cells consistent with primary neonatal microglia profile.	Gene expression data primarily from mouse iPSC-derived microglia. The human microglia model requires an isolation step. Majority of characterization done in mouse model and the system does not utilize neuronal cells. Not authentic YS ontogeny
Ormel et al. (110)	This protocol was adapted from Lancaster and Knoblich (111), with the only change made in media composition being increasing the concentration of Heparin (0.1 ug/ml to 1 ug/ml)	Characterizes innate development of microglia in hiPSC-derived brain organoids, which exhibit some phagocytic function as synaptic material is present within the cells.	Replication of these findings is currently lacking in the literature regarding the spontaneous differentiation of microglia in the organoid.
Haenseler et al. (10)	Utilizes IL-3 and M-CSF to drive myelopoiesis yielding a pure macrophage precursor population. Microglia differentiation and ramification of these cells is successfully induced using a neuronal base media (DMEM/F-12+N2 as a base media) + small molecules IL-34 and GM-CSF compared to X-VIVO which is used in the cultivation of monocytes and macrophages. The protocol utilizes X-VIVO and M-CSF for the maturation to macrophages as comparison.	Once set up, fully matured microglia can be generated at 2-week intervals for a 5-month period. Functional validation completed in a co-culture system. Only protocol to demonstrate a myeloblastosias proto-oncogene transcription factor (MYB)-independent YS origin using a MYB knockout iPSC line in previous work (112),	Requires a very sensitive 6–7-week period before microglia precursors can be collected. No assays showing functional integration into an animal model.

The precise protocol used however is likely to be dependent on the experimental question under investigation. YS, Yolk Sac. Additional references not in main text (104).

A second important question in the field, aside from debate around microglia ontogeny, is what phenotype should, microglia or microglia-like cells (MGLs) derived from hiPSC be considered “ideal”? As with ontogeny, considerable debate exists in the literature on this point. It may be considered that ultimately, the answer to this question depends on the nature of the scientific problem under investigation. For example, if one is studying the role of infiltrating macrophages upon injury, utilizing cells with brain-specific developmental ontogeny might not be necessary. Nonetheless, rational suggestions for what might constitute a “basic” work-up of hiPSC-derived MGLs in monoculture have recently been proposed (113). First, the protocol should ideally replicate an authentic, primitive YS ontogeny of human microglia, rather than following the haematopoietic lineage. Second, the cells generated should have a plausible microglia phenotype. That is to say, they should have a ramified morphology, express key surface markers (CD11b, CD45), proteins (Iba1, Tmem119, P2ry12, PU.1), and microglia signature genes (*MERTK, PROS1, GPR34, TMEM119* and so on), as well as any disease relevant genes of interest (57). The cells should also perform key microglial functions (including phagocytosis and secretion of cytokines in response to immune stimulation) as well as respond to adenosine triphosphate (ATP) stimulation *via* P2Y purinoceptor 12 (P2RY12) to produce intracellular calcium transients (57, 63, 113). Third, the protocol should be reproducible and reliable, within and between laboratories. On this point, there has been a limited effort to compare MGLs generated in any given protocol, to those generated by another, for example at the level of transcriptional profiling by RNA sequencing (115). A systematic comparison of such data across all protocols however, to the best of our knowledge, has yet to be performed. Clear cross-lab collaboration, data sharing, protocol comparisons, and communication are thus required in order to identify the best methods and small molecules for the differentiation of microglia from hiPSCs (10).

So far however, this only considers simple 2D monocultures of MGLs. This does offer the advantage of studying microglia phenotypes without interference from other cell types, as exemplified by recent work in the context of neurodegenerative diseases (63, 115). It may equally be argued however, that important phenotypic information is also lost due to the absence of interactions with other cell types including neurons and astrocytes, which is also required for evaluation of synaptic pruning (116). This has led to the development of more complex culture conditions, involving co-culture with hiPSC-derived neurons as 2D or 3D organoids (110). Of these, the 2D co-culture system carries some immediate advantages. It is simpler to implement and less heterogeneous than organoid models, particularly if the user combines MGLs with a homogenous population of forebrain excitatory neurons, for example generated through over-expression of Neurogenin-2 (Ngn2) (117). Furthermore, recent data from independent laboratories suggests that co-culture with neurons is necessary to produce microglia that are closer to a homeostatic, brain-resident phenotype (57, 105, 106, 108, 109). It would however be interesting to examine how embryonic macrophage progenitors, generated using the protocol of Haenseler and colleagues (57) migrate into brain organoids using “seeding” experiments (57). Nonetheless, the 2D neuron-microglia co-culture model also provides the useful opportunity to conduct match/mismatch experiments, whereby, the effect of patient-derived microglia on healthy control neurons may be assessed and vice versa for specific phenotypes. Sellgren and colleagues have previously successfully employed this type of experimental design for example, to demonstrate that factors intrinsic to PBMC derived iMG influence synaptic pruning independently of neurons (83). The 2D co-cultures are also amenable to high speed, multi-photon live imaging, essential for capturing, live, intricate interactions between microglia and neurons. For example, using gene-edited reporter lines from both healthy and SZ donors it would be interesting to examine any phenotypic differences in both synaptic density and connectivity within cultures, perhaps also using mix and mis-match experiments. Taking a different tack, if we accept that microglial heterogeneity is present in the human brain and that it is functionally important, it is reasonable to suggest that hiPSC microglia-neuron co-cultures could be used to investigate how this may arise, but also if this could be relevant in disease contexts. For example, one could imagine experiments to test whether co-culturing microglia with different hiPSC-derived neuronal cultures, such as cortical or ventral midbrain from the same donor, might influence human microglia form and function in a dish. This would offer the means to assess potential local cues and signalling mechanisms by which different neuronal populations might influence microglia function and vice versa, for example through conditioned media experiments. Such studies would also shed light on whether hiPSC *in vitro* models can recapitulate the diversity observed *in vivo* in human and rodent microglia, opening up further avenues for study, particularly in the context of brain disorders and at the same time, validating the model system further in comparison to human primary microglia.

Another key characteristic of an “ideal” hiPSC-microglia model is that it should be genetically modifiable. Advances in genome editing have rendered it potentially straightforward to assay genotype differences due to single gene mutations of disease relevant genes, with appropriate isogenic control lines as reported recently for Alzheimer’s disease (AD) relevant genetic variants using MGL monocultures (63, 115). This has, to the best of our knowledge yet to be extended to neuron-microglia co-culture models. In the context of modelling SZ and other neurodevelopmental disorders there are however some important considerations around the selection of the disease relevant mutation to investigate, to which we shall return later. For now however, there are some immediate genetic risk candidates that could be investigated. For example, the fractalkine receptor (CX3CR1), is of immediate interest based on *in vivo* findings previously mentioned in this review relating to synaptic pruning (78). Furthermore, rare single nucleotide polymorphisms in CX3CR1 are also associated with increased risk for SZ in humans (118). Importantly, the possibility that disruptions in microglia-mediated synaptic pruning *via* CX3CR1 could contribute to neurodevelopmental and neuropsychiatric disorders has yet to be tested in a human model system. Studies of microglia-neuron interactions using hiPSC models comparing individuals with genomic variation in Complement C4a would also likely be very informative based on existing genetic risk data for SZ and findings from iMG models (7, 83, 93). Here, mix/mis-match experiments could be highly applicable, for example, one could pair microglia with C4a or CX3CR1 risk variants with control neurons, or vice versa and study the effects on both synaptic density and neural connectivity (98). Studying the effect of rare CNVs that convey high risk for SZ may also be informative, for example in 22q11.2 deletion carriers.

As already alluded to, a key question in the context of modelling SZ is the choice of gene to study, since this is a highly polygenic disorder, with many common variants of small effect. Put another way, the mechanisms by which common risk variants of small effect interact to contribute to SZ pathophysiology is unclear. Schrode and colleagues (119) offer one solution to this problem, which is to use isogenic human hiPSC lines differentiated to neurons, to study the impact of SZ-associated common variants that are predicted to function as SZ expression quantitative trait loci (eQTLs) (119). Could a similar strategy however, be applied to microglia? Here one needs to consider data suggesting that the expression of common risk variants for SZ consistently maps onto pyramidal cells, medium spiny neurons, and specific interneurons, but not consistently to embryonic, progenitor, or glial cells, including microglia (120). These findings are in stark contrast to common risk variants for AD, which are enriched in microglia, among other cell types (120). This is consistent with the majority of hiPSC studies in SZ focussing to date on neuronal cells (98, 103, 119). However, this perhaps downplays the importance of studying non-neuronal cells using such models (103). For instance, it is conceivable that a gene could play a role in the pathophysiology of SZ, yet not be expressed in one of the “key” cell types implicated (120). Genetic polymorphisms in Complement C4a are a clear case in point, since there is clear evidence that C4a variants are involved in SZ neurobiology, yet the expression of C4a is high in microglia, but also in astrocytes and vascular leptomeningeal cells (93, 120). It may therefore be premature to exclude microglia (and other non-neuronal cells) from studies of how genetic risk variants for SZ affect their form and function. From a different perspective, one could argue that it would be relevant to examine how the neuronal phenotype induced by SZ-associated common variants may influence microglia phenotype, again using mix-match experiments. Such studies could be helpful to investigate if microglial pathology in SZ is a primary, causative, or secondary, responsive event (91). Another possible approach to circumvent this issue would be to generate hiPSC from individuals with either high or low polygenic risk score (PRS) for SZ and study how this influences microglia and/or microglia-neuron interactions in co-culture.

One other point worth noting here again is that SZ (and other neurodevelopmental psychiatric disorders) are thought to arise from a complex interaction between genetic and environmental risk factors. Hence, for hiPSC microglia models to truly reflect this model, studies of how environmental risk factors influence microglia form and function, but also neuron-microglia interactions are also essential. One such environmental risk factor that may be immediately amenable to such studies is MIA a known epidemiological risk factor for psychiatric disorders, including SZ, which we have already discussed in this review (121). As aforementioned, the hiPSC *in vitro* environment is easily manipulated. As such, the effect of pro-and anti-inflammatory cytokines elevated in response to MIA *in vivo*, or infectious pathogens that may cross the maternal-foetal interface, can easily be characterized to determine their influence on microglia activation states and/or neuron-microglia interactions. Precedent for such experiments comes from recent work examining how Zika virus for example, influences neuronal and glial phenotypes using a tri-culture hiPSC model system (116). Toll-like receptor 3 (TLR3) activation or application of single cytokines such as IL-6 could for example be used to phenocopy (to some extent) MIA *in vitro* using a human model system. This could then be compared to data from rodent models, for example the effect on microglia transcriptional profile and chromatin state, which is known to be abnormal early in development following MIA (122, 123). Such studies could be extended to examine how the inflammatory exposure might interact with specific genetic risk backgrounds, although this likely would be a complex undertaking.

A final advantage of neuron-microglia stem cell model systems is that drug screening may also easily be performed in combination with high content imaging or other high-throughput assays. As an indicative example, Sellgren et al. (83) demonstrated a reduced engulfment of synaptic material by iMG following treatment with the broad-spectrum anti-inflammatory agent minocycline (83).

A further key limitation however, aside from the key questions regarding microglia ontogeny and phenotype generated between different hiPSC-microglia protocols, is that human primary microglia display major differences in morphology and gene expression when grown in culture, including down-regulation of signature microglial genes (39). Hence, a case may be made that *in vitro* microglia derived from hiPSC may not accurately represent resting human primary microglia. This only serves to confirm the importance of the context in which the hiPSC microglia are maintained, for example, with or without neurons in co-culture and so on (113). In support of this view, transcriptomic studies provide evidence to suggest that hiPSC-derived microglia when co-cultured with neurons align with foetal human primary microglia and do express key microglia signature genes, even in monocultures (57, 63, 105–109, 115). On the other hand, Abud and colleagues (106) compared their hiPSC microglia to both foetal and adult human primary microglia using transcriptional profiling revealing more than 2,000 genes with increased expression in hiPSC microglia as compared to foetal microglia and >1,000 genes as compared to adult microglia (106, 113). Hence, clearly further work is required to determine how comparable hiPSC-derived microglial cells are with either early or late primary human microglia.

One possibility to circumvent this issue is the potential for transplanting hiPSC-derived microglial precursors into adult rodent brains to create chimeric model systems (124, 125). Excitingly, this has also been recently demonstrated using neonatal mice, as young as postnatal day 0 (38, 126). Importantly, in both adult and neonatal rodent brains, the hiPSC-derived microglial precursors integrated successfully and acquired characteristic microglial morphologies and gene expression signatures, closely resembling that of human primary microglia (38, 124–126). Single cell RNA sequencing analysis confirmed the presence of cellular transcriptional heterogeneity in the implanted hiPSC-microglia (38, 126), consistent with observations in human primary microglia (49). These chimeric models provide powerful new tools for interrogating species-specific differences between human and rodent microglia at molecular, functional, and behavioral levels (see **Table 1**). Moreover, since transplantation into the neonatal brain is feasible, studies of the processes in which microglia are intimately involved during neuronal development, such as neurogenesis, synaptogenesis, and synaptic pruning is rendered possible for the first time using human microglia in host brain environment (38). Such models will therefore be useful to investigate how human and mouse microglia function differently in shaping neuronal development using combination of “omics” tools and *in vivo* 2-photon imaging (38). Moreover, the chimeric model approach enables *in vivo* studies of how microglia derived from individuals with different psychiatric diagnosis differ from those of otherwise healthy donors. For example, it would be fascinating to study how microglia derived from individuals with SZ with high or low polygenic risk profiles, shape neurodevelopment *in vivo* (124, 127). This should include, for example, *in vivo* imaging studies of microglia-synapse interactions to complement the observations made *in vitro* by Sellgren and colleagues (7, 83). As already mentioned, the transplantation procedure means that the microglia express a transcriptomic signature that is much closer to *in vivo* human microglia, as compared to culture models, even co-culture or organoid-models, although how much this influenced by the age of the host remains to be characterized in depth (38, 124, 127). Collectively then, experiments done using patient derived microglia and neurons *in vitro* may be complemented by parallel *in vivo* studies using chimeric models, which will likely improve the chances of results from such studies translating into effective human treatments (126). There remain however important limitations to this technique that still need to be overcome. For example, whilst microglia can be generated from a variety of patient hiPSC lines in a straightforward manner (57), mice expressing humanized forms of key microglia survival factors such as IL-34 and CSF-1 must be used in order for the xenograft transplantation of hiPSC-derived microglia to be successful (124, 127). Other limitations that remain to be addressed include concerns related to the effect of the murine host cells on the functionality of the xenotransplanted human microglia. For example, there is only limited homology between several mouse and human proteins and the downstream effects such differences may have on cell-to-cell interactions and microglia activation/inhibition is unknown (127). The response of the host microglia is also a potential confounding factor (127) and reliable results may depend on depletion of these cells using chemical ablation (128) or mice that lack endogenous microglia (129). Finally, whilst such chimeric models could be used to study the influence environmental risk factors for SZ using human xenografted microglia, including MIA, it should be remembered that the host mice are immune-deficient, which may be a confounding factor in the response to a systemic immune stimulus (127). Nonetheless, the potential for chimeric models to facilitate our understanding of neuron-microglia interactions in relation to SZ and other neurodevelopmental disorders is clear.

## Conclusions

At present, we are in the early stages of understanding of microglia in both health and disease including potential functional consequences of microglia heterogeneity. The use of several models is essential to replicate and translate findings to humans from rodent models. The hiPSC system offers a human-specific model with the potential to study a diverse population of microglia either as monocultures or in co-culture with defined neuronal (and other non-neuronal) cells. Whilst there are many advantages to this system that could be applied to studying the role of microglia in psychiatric disorders with a neurodevelopmental origin, there are also key challenges for the field to overcome. Specifically, questions and debate remain over the precise differentiation protocol to use, particularly with regard to the question of what constitutes “authentic” microglia ontogeny. Furthermore, how the field should define what constitutes an “ideal” microglial phenotype is also far from clear. Concerns regarding the similarity between hiPSC microglia and human primary microglia are also on going, although chimeric models offer one exciting new direction to address this question. In addition, progress is being made on several fronts to address the other concerns, including rational suggestions for phenotypic workup of hiPSC-derived microglia (113). Data sharing between laboratories is also critical to address potential questions around reliability and reproducibility. Nevertheless, we judge that there is sufficient evidence to suggest that hiPSC-derived microglia-neuron co-culture models have great potential as a human *in vitro* model system with which to test key hypotheses related to neuro-immune interactions and the pathogenesis of psychiatric disorders with a neurodevelopmental origin, including SZ.

## Author Contributions

BH: conception and design, literature searching, manuscript writing. AC: manuscript writing and generation of **Figure 1**. LR: manuscript writing and editing. DS: manuscript writing, editing, and financial support. AV: conception and design, manuscript writing, financial support, final approval of manuscript.

## Conflict of Interest

The authors declare that the research was conducted in the absence of any commercial or financial relationships that could be construed as a potential conflict of interest.
